# Improving chlamydia knowledge should lead to increased chlamydia testing among Australian general practitioners: a cross-sectional study of chlamydia testing uptake in general practice

**DOI:** 10.1186/s12879-014-0584-2

**Published:** 2014-11-07

**Authors:** Anna Yeung, Meredith Temple-Smith, Simone Spark, Rebecca Guy, Christopher K Fairley, Matthew Law, Anna Wood, Kirsty Smith, Basil Donovan, John Kaldor, Jane Gunn, Marie Pirotta, Rob Carter, Jane Hocking

**Affiliations:** Centre for Epidemiology and Biostatistics, Melbourne School of Population and Global Health, University of Melbourne, 207 Bouverie Street, Carlton, Victoria Australia; General Practice and Primary Health Care Academic Centre, University of Melbourne, 200 Berkeley Street, Carlton, Victoria Australia; The Kirby Institute, Wallace Wurth Building, University of New South Wales, Sydney, New South Wales Australia; Melbourne Sexual Health Centre, Alfred Health, 580 Swanston Street, Carlton, Victoria Australia; Central Clinical School, Monash University, Alfred Hospital, 55 Commercial Road, Melbourne, Victoria Australia; Sydney Sexual Health Centre, Sydney Hospital, 8 Macquarie Street, Sydney, NSW Australia; School of Health and Social Development, Deakin University, 221 Burwood Highway, Burwood, Victoria Australia; the ACCEPt consortium, Australia

**Keywords:** Chlamydia testing, General practice, Sexual health knowledge, General practitioner education

## Abstract

**Background:**

Female general practitioners (GPs) have higher chlamydia testing rates than male GPs, yet it is unclear whether this is due to lack of knowledge among male GPs or because female GPs consult and test more female patients.

**Methods:**

GPs completed a survey about their demographic details and knowledge about genital chlamydia. Chlamydia testing and consultation data for patients aged 16-29 years were extracted from the medical records software for each GP and linked to their survey responses. Chi-square tests were used to determine differences in a GP’s knowledge and demographics. Two multivariable models that adjusted for the gender of the patient were used to investigate associations between a GP and their chlamydia testing rates ― Model 1 included GPs’ characteristics such as age and gender, Model 2 excluded these characteristics to specifically examine any associations with knowledge.

**Results:**

Female GPs were more likely than male GPs to know when to re-test a patient after a negative chlamydia test (18.8% versus 9.7%, p = 0.01), the correct symptoms suggestive of PID (80.5% versus 67.8%, p = 0.01) and the correct tests for diagnosing PID (57.1% versus 42.6%, p = 0.01). Female GPs tested 6.5% of patients, while male GPs tested 2.2% (p < 0.01). Model 1 found factors associated with chlamydia testing were being a female GP (OR = 2.5, 95% CI: 1.9, 3.3) and working in a metropolitan clinic (OR = 3.2; 95% CI: 2.4, 4.3). Model 2 showed that chlamydia testing increased as knowledge of testing guidelines improved (3-5 correct answers – AOR = 2.0, 95% CI: 1.0, 4.2; 6+ correct answers – AOR = 2.9, 95% CI: 1.4, 6.2).

**Conclusions:**

Higher rates of chlamydia testing are strongly associated with GPs who are female, based in a metropolitan clinic and among those with more knowledge of the recommended guidelines. Improving chlamydia knowledge among male GPs may increase chlamydia testing.

**Electronic supplementary material:**

The online version of this article (doi:10.1186/s12879-014-0584-2) contains supplementary material, which is available to authorized users.

## Background

Chlamydia is the most common bacterial sexually transmissible infection (STI) in Australia; notifications have risen dramatically over the past decade, from 30 000 in 2003 to more than 80 000 notifications in 2013 [1]. Over 80% of infections in both men and women are asymptomatic [2] and if left untreated, chlamydia can cause pelvic inflammatory disease (PID) and progress to infertility in women [3],[4].

In Australia, 80% of chlamydia cases are diagnosed in the general practice setting, with the other 20% coming from sexual health or family planning clinics, hospitals and other specialist services [5]. The current Royal Australian College of General Practitioners (RACGP) Preventive Care Guidelines recommends annual testing for all sexually active young people aged 15-29 years [6]. Although 75% of young people in this age group are seen by general practitioners (GP) at least once a year [7], testing rates remain below 10%. Modelling data has shown that testing rates need to be increased to 30% or more in order to reduce the prevalence of chlamydia [8]. Barriers to testing by GPs include lack of time, discomfort with sexual health issues [9] and lack of knowledge about the benefits of chlamydia screening [10].

Higher self-reported chlamydia testing rates have been found to be associated with being a female GP [11] or having postgraduate qualifications in sexual health [12] but the association between empirically measured chlamydia testing rates and a GP’s knowledge has not been investigated. An audit of testing data in general practice also found female GPs and GPs practicing in a major city are more likely to test for chlamydia [13] but this study did not consider the influence of GPs’ knowledge about chlamydia diagnosis and management.

We had the opportunity to investigate associations between GP gender, their knowledge and their chlamydia testing rates in an ongoing chlamydia testing trial in general practice. As part of this trial, GPs were asked to complete a survey about their knowledge of chlamydia diagnosis and management. Survey responses were subsequently correlated with their chlamydia testing data for the 12 months prior to commencing the trial. This paper presents these results and examines whether a GP’s knowledge varies by GP gender and age, and whether a GP’s demographic characteristics and sexual health knowledge is associated with their chlamydia testing rates.

## Methods

### Setting

The Australian Chlamydia Control Effectiveness Pilot (ACCEPt) is a cluster randomised controlled trial that aims to determine whether a chlamydia testing intervention in general practice can lead to a reduction in chlamydia prevalence in the population [14]. ACCEPt is using a cluster design to reduce the likelihood of patients attending both participatory and non-participatory clinics. A total of 134 general practice clinics and Aboriginal medical services in 54 rural and regional towns (clusters) in the Australian states of Victoria, New South Wales, Queensland and South Australia are enrolled in ACCEPt. A list of towns was drawn up and towns were selected in no particular order until the required sample size of 54 was reached. A further nine general practice clinics from metropolitan areas in Victoria were also recruited. Recruitment took place between mid 2010 and December 2011. All GPs at each participating clinic were consented into ACCEPt.

### General practice in Australia

Primary care in Australia is largely provided by general practice clinics and patients can choose to attend multiple different clinics, as opposed to the United Kingdom where patients are required to register with one practice. It is not guaranteed that a patient will always see the same GP in each clinic, as this depends on GP availability and the patient’s preference.

### Questionnaire administration

On enrolment of the clinic, each GP was asked to complete a survey on their knowledge of the diagnosis and management of chlamydia. The GP was asked to return the paper survey to the researcher or mail it to the Melbourne School of Population and Global Health at the University of Melbourne. GPs were also consented to allow their chlamydia testing and consultation data to be extracted from the medical records software at the clinic. Data from the survey were entered into a Microsoft Access database.

### Questionnaire content and format

The survey was adapted from previous chlamydia survey studies conducted by the University of Melbourne [10]; face and content validity was established by experts in the field and then pilot tested with GPs. The survey included demographic questions (age, sex, number of years working in general practice, postgraduate qualifications), knowledge based questions about age groups at greatest risk of chlamydia, clinical presentation of chlamydia, antibiotics for treating chlamydia, follow-up testing recommendations and diagnosis and management of PID. A variety of testing scenarios asked the GP to indicate how likely they would be to offer a chlamydia test to the patient presenting in the scenario (for instance, an 18 year old woman presenting with low abdominal pain) to determine their knowledge about the recommended testing guidelines. The majority of questions used a Likert scale format to measure the GP’s response. Knowledge based questions used a mix of multiple choice and free-text space. Such questions aimed, for example, to determine the appropriate follow-up time after a positive or negative chlamydia test.

Answers to the questions about epidemiology, clinical presentation, antibiotic treatment and follow-up testing were based on data from the National Notifiable Disease Surveillance System, the RACGP guidelines, the Centers for Disease Control and Prevention guidelines and the National Management Guidelines for Sexually Transmissible Infections, as well as input from two clinicians from the ACCEPt investigator team on PID diagnosis. At the time of the survey, the RACGP guidelines recommended testing all sexually active young people between 15 and 25 [15] and the survey was based on these guidelines. However in 2012, revised guidelines were released recommending testing young people up to 29 years of age annually [6]. This analysis was based on the RACGP guidelines available in 2010.

### Chlamydia testing data

A data extraction tool (Grhanite™) was installed on a computer in each clinic to extract medical record data from the clinic’s patient records system. Further information about Grhanite™ is provided by Boyle et al. [16] and is available on the website [17]. This tool extracts de-identified patient data about all consultations including GP provider number (a unique GP identification code), date of consultation, age and sex of patient, whether the patient had a chlamydia test and the result of the test. The chlamydia testing data obtained from clinics using Grhanite™ against laboratory chlamydia testing data has been previously validated [18]. Data were extracted weekly from each clinic. For each GP who completed the survey, the number of patients aged 16 to 29 years who had at least one chlamydia test was divided by the number of patients who had consulted that particular GP at least once during 2010. These data were used to calculate an individual GP’s chlamydia testing rate among patients aged 16 to 29 years for 2010, and while we refer to it as a chlamydia testing `rate’ throughout this manuscript, it is actually a measure of patients who had a chlamydia test proportionate to patients who had a consultation. To be included in the analysis presented in this paper, a GP had to have worked at the participating clinic for at least one month in 2010 and completed the survey at the time of enrolment.

### Data analysis

Each GP’s chlamydia testing data were linked to their survey data by a unique study number and analysed in Stata™ (College Station, Texas). Chi-square tests were used to examine differences between male and female GPs in their demographic details and their knowledge about chlamydia diagnosis and management. The questions using a Likert scale format were coded into binary answers (correct/incorrect or yes/no), with `don’t know’ or `not sure’ answers included in the no or incorrect category. Free-text answers were also coded into either correct or incorrect categories. Each of the eight testing scenarios was coded as a binary answer (offer test/not offer test) and then classified as either correct or incorrect testing practices according to the 2010 RACGP chlamydia testing guidelines. The total number of correct testing practices was then summed for each GP to get a testing score out of eight. To evaluate knowledge about the diagnosis and management of PID, GPs were asked to indicate which signs and symptoms were suggestive of PID from a list. They were classified as answering correctly if they selected each of the following - tenderness with motion of the cervix, adnexal tenderness, uterine tenderness, lower abdominal tenderness and inflamed cervix. They were also asked to choose which tests could be used to help diagnose PID in a general practice setting and were classified as answering correctly if they selected each of the following - pregnancy test, test for chlamydia and gonorrhoea, abdominal palpation and bimanual examination.

Logistic regression was used to assess associations between each GP’s chlamydia testing rates and their demographics and knowledge about the diagnosis and management of chlamydia, and odds ratios and 95% confidence intervals were calculated. An individual GP’s chlamydia testing rate was included in the model as a binomial proportion where the numerator was the total number of patients who had at least one chlamydia test and the denominator was the total number of patients who had at least one consultation. All analyses were conducted using Stata 13 (College Station, Texas) and used a generalised linear modelling command with a logit link function and binomial family; both unadjusted and multivariable analyses were undertaken. Initial analysis showed that female GPs had greater knowledge about chlamydia and that being a female GP was the variable most strongly predictive of chlamydia testing. To enable us to examine the association of knowledge with chlamydia testing, we present two multivariable models. Model 1 included both the characteristics of the GP – age, sex, location and interest in sexual health – and knowledge variables. Variables that had a significant association (p < 0.1) with testing in the unadjusted analysis were included in the multivariable analysis. Model 2 excluded the individual characteristics of the GP but included all knowledge variables. Patient gender was included in both Model 1 and Model 2 because previous work has shown that female GPs are more likely to see female patients [11] and testing rates are higher among female patients than male patients [7].

The survey received ethical approval from the Royal Australian College of General Practitioners Ethics Committee, the University of Melbourne Human Research Ethics Committee and the Aboriginal Health and Medical Research Council.

## Results

### Response rates

A total of 457 GPs had chlamydia testing data available in 2010 and of these, 391 had completed a survey, resulting in a response rate of 85.6%.

### Participants

The majority of GPs were male (66.0%), and most GPs were trained in Australia (63.3%). Over half were aged between 45-59 years (52.9%) with less than 5% being under 30 years of age (Table 1). The number of years working in general practice averaged 17.2 years (interquartile range: 6 - 26). Female GPs were more likely to be younger, to work fewer sessions per week and to have an interest in sexual health than male GPs (all p < 0.01).

**Table 1:** **Characteristics of participating GPs**

Characteristic		Overall N (%)	Male GP N (%)	Female GP N (%)	p-value
Gender of GP			258 (66.0)	133 (34.0)	<0.01
Location of GP clinic	Rural	346 (88.5)	234 (90.7)	112 (84.2)	0.06
	Metropolitan	45 (11.5)	24 (9.3)	21 (15.8)	
GP Age Group (years)	<30	13 (3.3)	6 (2.3)	7 (5.3)	<0.01
	30-44	124 (31.7)	70 (27.1)	54 (40.6)	
	45-59	207 (52.9)	143 (55.4)	64 (48.1)	
	>60	47 (12.0)	39 (15.1)	8 (6.0)	
Years working in general practice	<5	74 (19.2)	41 (16.1)	33 (25.0)	<0.01
	5-10	40 (10.4)	18 (7.1)	22 (16.7)	
	10-20	89 (23.1)	57 (22.4)	32 (24.2)	
	20-30	117 (30.3)	83 (32.7)	34 (25.8)	
	30+	66 (17.1)	55 (21.7)	11 (8.3)	
Country of medical training	Australia	247 (63.3)	161 (62.7)	86 (64.7)	0.70
	Overseas	143 (36.7)	96 (37.4)	47 (35.3)	
Number of clinic sessions per week	<5	35 (9.2)	11 (4.4)	24 (18.5)	<0.01
	5-9	267 (70.3)	176 (70.4)	91 (70.0)	
	10+	78 (20.5)	63 (25.2)	15 (11.5)	
Postgraduate qualifications^1^	No	117 (29.9)	75 (29.1)	42 (31.6)	0.61
	Yes	274 (70.1)	183 (70.9)	91 (68.4)	
Interest in sexual health	No	284 (73.8)	207 (80.9)	77 (59.7)	<0.01
	Yes	101 (26.2)	49 (19.1)	52 (40.3)	

^1^Qualifications include Diploma of Obstetrics & Gynaecology, Diploma of Venereology/Sexual Health, Certificate of the Family Planning Association, Fellow of the Royal Australian and New Zealand College of Obstetricians and Gynaecologists, Fellow of the Royal Australian College of General Practitioners, Fellow of the Australian College of Rural and Remote Medicine.

### Knowledge

While over 90% of GPs knew infection in women was usually asymptomatic, only 74% knew infection in men was also usually asymptomatic (Table 2). Over 80% knew the correct treatment for non-complicated chlamydia infection, but only about 40% knew that azithromycin should be used as the first line treatment for chlamydia during pregnancy. Knowledge about correct re-testing intervals after either a negative test (12 months) or a positive test result (3 months) was poor, 12.8% and 21.2% respectively. Female GPs were more likely than male GPs to indicate that they would conduct a chlamydia test for a 23 year old woman presenting for a pap smear (66.9% versus 46.9%, p < 0.01) and according to the RACGP Guidelines in 2010, more likely to incorrectly test a 26 year old man presenting for a truck license (26.3% versus 15.6%, p = 0.01) and a 33 year old woman presenting for an oral contraceptive prescription (21.1% versus 12.1%, p = 0.02).

**Table 2:** **Knowledge about chlamydia diagnosis and management by GP gender**

Variable		Overall N (%)	Male GP N (%)	Female GP N (%)	p-value
Female age groups at highest risk of infection^1^	Incorrect	24 (6.3)	16 (6.4)	8 (6.1)	0.91
	Correct	244 (93.8)	236 (93.7)	124 (93.9)	
Male age groups at highest risk of infection^2^	Incorrect	39 (10.4)	24 (9.8)	15 (11.5)	0.63
	Correct	336 (89.6)	220 (90.2)	116 (88.6)	
Chlamydia is usually asymptomatic in Women	Disagree	32 (8.3)	20 (7.9)	12 (9.0)	0.70
	Agree	355 (91.7)	234 (92.1)	121 (91.0)	
Chlamydia is usually asymptomatic in Men	Disagree	100 (26.0)	67 (26.6)	33 (24.8)	0.71
	Agree	285 (74.0)	185 (73.4)	100 (75.2)	
Knowledge of population groups to be targeted for screening^3^ *(see below for results for each scenario)*	0-2	24 (6.2)	19 (7.5)	5 (3.8)	0.09
	3-5	264 (68.6)	177 (70.2)	87 (65.4)	
	6+	97 (25.2)	56 (22.2)	41 (30.8)	
Treatment in men and non-pregnant women^4^	Incorrect	70 (17.9)	49 (19.0)	21 (15.8)	0.43
	Correct	321 (82.1)	209 (81.0)	112 (84.2)	
Treatment in pregnant women^4^	Incorrect	231 (59.1)	146 (56.6)	85 (63.9)	0.16
	Correct	160 (40.9)	112 (43.4)	48 (36.1)	
Retest 12 months after a negative test^5^	Incorrect	341 (87.2)	233 (90.3)	108 (81.2)	0.01
	Correct	50 (12.8)	25 (9.7)	25 (18.8)	
Retest 3 months after a positive test^5^	Incorrect	308 (78.8)	207 (80.2)	101 (75.9)	0.33
	Correct	83 (21.2)	51 (19.8)	32 (24.1)	
Knowledge of symptoms suggestive of PID^6^	Incorrect	109 (27.9)	83 (32.2)	26 (19.6)	0.01
	Correct	282 (72.1)	175 (67.8)	107 (80.5)	
Knowledge of PID tests that should be done^7^	Incorrect	205 (52.4)	148 (57.4)	57 (42.9)	0.01
	Correct	186 (47.6)	110 (42.6)	76 (57.1)	
Testing Scenarios					
Case 1: 23 year old female, pap smear	Not offer test	180 (46.3)	136 (53.1)	44 (33.1)	<0.01
	Offer test	209 (53.7)	120 (46.9)	89 (66.9)	
Case 2: 18 year old female, abdominal pain	Not offer test	19 (4.9)	16 (6.2)	3 (2.3)	0.08
	Offer test	371 (95.1)	241 (93.8)	130 (97.7)	
Case 3: 26 year old male, truck license medical	Not offer test	315 (80.8)	217 (84.4)	98 (73.7)	0.01
	Offer test	75 (19.2)	40 (15.6)	35 (26.3)	
Case 4: 24 year old female, 16/40 pregnant	Not offer test	228 (58.8)	151 (59.2)	77 (57.9)	0.80
	Offer test	160 (41.2)	104 (40.8)	56 (42.1)	
Case 5: 22 year old male, Aboriginal, sore throat	Not offer test	271 (69.5)	178 (69.3)	93 (69.9)	0.89
	Offer test	119 (30.5)	79 (30.7)	40 (30.1)	
Case 6: 33 year old female, pill script	Not offer test	330 (84.8)	225 (87.9)	105 (79.0)	0.02
	Offer test	59 (15.2)	31 (12.1)	28 (21.1)	
Case 7: 17 year old male, genital warts	Not offer test	12 (3.1)	11 (4.3)	1 (0.8)	0.06
	Offer test	378 (96.9)	246 (95.7)	132 (99.3)	
Case 8: 34 year old male, 2 partners in last 6 months, HIV test	Not offer test	10 (2.6)	6 (2.3)	4 (3.0)	0.70
	Offer test	379 (97.4)	250 (97.7)	129 (97.0)	

^1^Answers were classified as correct if they ticked at least one correct answer - 15-19 and 20-24 year olds; ^2^Answers were classified as correct if they ticked at least one correct answer - 20-24 and 25-29 year olds; ^3^Knowledge of population groups to be targeted for screening is one point for correctly offering a test under the 2010 RACGP guidelines - tests should be offered to cases 1, 2, 4, 5, 7 & 8 but not offered to cases 3 & 6; ^4^Azithromycin is the correct treatment for men, women and pregnant women; ^5^RACGP guidelines recommend re-testing every 12 months after a negative test and every 3 months after a positive test; ^6^Correct signs and symptoms suggestive of PID are tenderness with motion of the cervix, adnexal tenderness, uterine tenderness, lower abdominal tenderness and inflamed cervix; ^7^Correct diagnostic tests are pregnancy test, chlamydia and gonorrhoea test, abdominal palpation and bimanual examination.

Female GPs were more likely than male GPs to know when to re-test a patient after a negative chlamydia test (18.8% versus 9.7%, p = 0.01), were more likely to correctly know the symptoms suggestive of PID (80.5% versus 67.8%, p = 0.01) and which tests should be used for diagnosing PID (57.1% versus 42.6%, p = 0.01). No other significant differences in knowledge between male and female GPs were observed.

### Testing rates

The overall testing rate in 2010 was 3.7% (95% CI: 2.9%, 4.4%) for 16-29 year old patients. Female GPs had a higher testing rate in 2010 of 6.5% (95% CI: 5.1%, 7.9%) than male GPs (2.2%; 95% CI: 1.4%, 3.0%; p < 0.01). In addition, younger GPs also had a higher testing rate irrespective of the gender of the GP; GPs aged less than 30 years tested 6.5% (95% CI: 4.2%, 8.9%) while GPs aged 60 and over tested 2.3% (95% CI: 0.6%, 4.0%) of 16-29 year olds. Those practicing in rural areas had a lower testing rate of 5.5% (95% CI: 4.7%, 6.2%) than those practicing in metropolitan areas (9.0%; 95% CI: 6.5%, 11.6%).

### Factors associated with chlamydia testing

Multivariable model 1 showed that being a female GP (Adjusted OR [AOR] =2.5; 95% CI: 1.9, 3.3), having an interest in sexual health (AOR = 1.3, 95% CI: 1.0, 1.7), and working in a metropolitan area (AOR = 3.2; 95% CI: 2.4, 4.3) were associated with an increased odds of testing. An increase in the age of the GP was associated with lower rates of testing (45-59 years old: AOR = 0.5, 95% CI: 0.3, 0.7; 60+ years old: AOR = 0.4, 95% CI: 0.3, 0.7; relative to GPs aged <30). No knowledge variables were independently associated with chlamydia testing (Table 3).

**Table 3:** **GP characteristics and knowledge variables associated with chlamydia testing** ^**1**^

GP variable		Odds ratio	95% CI	p-value	Adjusted OR^2^(including GP characteristics)	95% CI	p-value	Adjusted OR^2^(excluding GP characteristics)	95% CI	p-value
Gender of GP	Male	1.0			1.0					
	Female	3.1	2.1, 4.7	<0.01	2.5	1.9, 3.3	<0.01			
Location	Rural	1.0			1.0					
	Metro	2.3	1.4, 3.6	<0.01	3.2	2.4, 4.3	<0.01			
Age Group	<30	1.0			1.0					
	30-44	0.8	0.5, 1.2	0.27	0.7	0.5, 1.1	0.13			
	45-59	0.6	0.3, 0.9	0.02	0.5	0.3, 0.7	<0.01			
	60+	0.4	0.1, 1.3	0.13	0.4	0.3, 0.7	<0.01			
Years in general practice	<5	1.0								
	5-10	1.0	0.6, 1.7	0.91						
	10-20	0.9	0.6, 1.5	0.81						
	20-30	0.8	0.4, 1.5	0.48						
	30+	0.4	0.1, 1.3	0.11						
Country of training	Australian	1.0								
	trained									
	Overseas trained	0.8	0.5, 1.2	0.31						
Interest in sexual health	No	1.0			1.0					
	Yes	1.7	1.0, 2.7	0.04	1.3	1.0, 1.7	0.03			
Postgraduate qualifications	No	1.0								
	Yes	1.2	0.7, 1.9	0.56						
Female age groups at highest risk of infection^3^	Incorrect	1.0			1.0			1.0		
	Correct	1.8	1.0, 3.1	0.05	1.6	0.7, 3.5	0.26	1.5	0.7, 3.5	0.31
Male age groups at highest risk of infection^4^	Incorrect	1.0			1.0			1.0		
	Correct	1.6	0.9, 2.9	0.08	1.4	0.8, 2.7	0.23	1.6	0.9, 2.9	0.10
Chlamydia is usually asymptomatic in Women	Disagree	1.0						1.0		
	Agree	1.4	0.9, 2.3	0.18				1.2	0.7, 2.1	0.44
Chlamydia is usually asymptomatic in Men	Disagree	1.0			1.0			1.0		
	Agree	1.6	1.1, 2.2	0.01	1.4	1.0, 1.9	0.03	1.4	0.9, 1.9	0.10
Knowledge of population groups to be targeted for screening^5^	0-2	1.0			1.0			1.0		
	3-5	2.0	1.0, 4.0	0.05	1.7	0.8, 3.8	0.16	2.0	1.0, 4.2	0.07
	6+	3.4	1.7, 6.8	<0.01	2.0	0.9, 4.4	0.08	2.9	1.4, 6.2	<0.01
Treatment in men and non-pregnant women^6^	Incorrect	1.0						1.0		
	Correct	1.0	0.4, 2.3	0.97				1.0	0.5, 2.2	0.92
Treatment in pregnant women^6^	Incorrect	1.0						1.0		
	Correct	0.7	0.5, 1.0	0.11				0.6	0.4, 0.9	0.01
Retest at 12 months after a negative test^7^	Incorrect	1.0			1.0			1.0		
	Correct	1.8	0.9, 3.5	0.08	1.2	0.9, 1.6	0.32	1.5	0.9, 2.6	0.16
Retest at 3 months after a positive test^7^	Incorrect	1.0						1.0		
	Correct	1.0	0.7, 1.5	0.97				0.9	0.6, 1.4	0.73
Knowledge of symptoms suggestive of PID^8^	Incorrect	1.0						1.0		
	Correct	1.3	0.7, 2.2	0.38				1.3	0.8, 2.0	0.34
Knowledge of PID tests that should be done^9^	Incorrect	1.0			1.0			1.0		
	Correct	1.5	0.9, 2.3	0.09	0.9	0.7, 1.2	0.41	1.1	0.8, 1.7	0.51
Gender of patient^2^	Male	1.0			1.0			1.0		
	Female	2.3	1.7, 3.1	<0.01	1.9	1.5, 2.4	<0.01	2.2	1.7, 2.9	<0.01

^1^Accounted for repeated measures from individuals GPs; ^2^The multivariable models have adjusted for patient gender; ^3^Answers were classified as correct if they ticked at least one correct answer - 15-19 and 20-24 year olds; ^4^Answers were classified as correct if they ticked at least one correct answer - 20-24 and 25-29 year olds; ^5^Knowledge of population groups to be targeted for screening is one point for correctly offering a test under the 2010 RACGP guidelines - tests should be offered to cases 1, 2, 4, 5, 7 & 8 but not offered to cases 3 & 6; ^6^Azithromycin is the correct treatment for men, women and pregnant women; ^7^RACGP guidelines recommend re-testing every 12 months after a negative test and every 3 months after a positive test; ^8^Correct signs and symptoms suggestive of PID are tenderness with motion of the cervix, adnexal tenderness, uterine tenderness, lower abdominal tenderness and inflamed cervix; ^9^Correct diagnostic tests are pregnancy test, chlamydia and gonorrhoea test, abdominal palpation and bimanual examination.

Multivariable model 2 showed that GPs with increasing knowledge of the correct testing guidelines were more likely to test (3-5 correct answers – AOR =2.0, 95% CI: 1.0, 4.2; 6+ correct answers – AOR = 2.9; 95% CI: 1.4, 6.2; relative to those with 0-2 correct answers). GPs who knew the correct treatment for chlamydia in pregnant women had lower odds of testing (AOR = 0.6, 95% CI: 0.4, 0.9). No other factors were independently associated with chlamydia testing (Table 3).

## Discussion

Our analysis is the first to link GPs’ chlamydia testing rates with their knowledge about chlamydia and to examine the association of knowledge with GP gender. We found the strongest independent predictors of chlamydia testing were being a female GP and being based in a metropolitan clinic. When the individual GP characteristics were excluded from the multivariable model, we found that GPs with knowledge of who should be tested according to the RACGP guidelines had increasing chlamydia testing rates.

The strengths of this study include its high response rate of over 85%, representation of GPs from four Australian states (Victoria, New South Wales, Queensland and South Australia), and that gender of patient was controlled for in the analysis. Controlling for gender of patient was important because testing rates are higher in female than male patients [7] and female GPs are more likely than male GPs to consult with female patients [19]. However, a limitation of our analysis was that over 80% of GPs were from rural clinics, leading to an underrepresentation of GPs working in metropolitan clinics. Given that GPs working in metropolitan clinics were more likely to have higher chlamydia testing rates, the results of our study may not be representative of GPs overall.

In this study, female GPs had significantly greater knowledge than male GPs about who should be tested for chlamydia, when to conduct repeat testing after a negative test and the diagnosis and management of PID. Additionally, GPs with increased knowledge about who should be tested for chlamydia had higher testing rates. This suggests that if we can increase GP knowledge about the diagnosis and management of chlamydia, chlamydia testing rates should increase. While continuing medical education is frequently cited as a facilitator to increased chlamydia testing [10], national leadership is needed to improve the quality and effectiveness of sexual health education for GPs, particularly male GPs. Given that females make up only 41% of the general practice workforce in Australia [20] and there are fewer female GPs available in rural areas than metropolitan areas, the results of our study show that addressing these gender differences in sexual health knowledge is imperative to improve chlamydia testing access for all young adults across Australia.

It is likely that the strong association between chlamydia testing and female GPs is ingrained in the culture of general practice. Firstly, females are more likely to attend general practice than males [21] and they are more likely to attend for a sexual health related consultation such as contraceptive advice and pap smears, contexts where an offer of a chlamydia test is easier [22]. Secondly, as the consequences of untreated chlamydia have a greater impact on women, it is possible that female GPs are more conscious about the importance of chlamydia testing [23]. Burd and colleagues investigated the impact of physician gender on sexual history taking in the USA and found that male physicians were more likely than female physicians to report discomfort in taking a sexual history from a female patient (35% versus 12%) and to perceive that female patients were uncomfortable with having their sexual history taken (53% versus 24%) [24] which may impact their chlamydia testing rates. We also found that testing rates decreased with increasing age of the GP, although this may be due to patients self-selecting younger doctors for STI testing. Data show that younger patients prefer a younger female GP because they consider them to be less conservative and easier to relate to than older GPs [25]. Conversely, older GPs with an older patient base may be less mindful of chlamydia testing and use guidelines less frequently [26].

It is possible that receiving a positive chlamydia test result for a patient may motivate a GP to test more as previous research has shown that GPs who reported testing symptomatic patients weekly were more likely to also test asymptomatic patients compared to GPs who tested symptomatic patients less often [11]. We were unable to investigate the effect of positive results in this analysis because of the cross-sectional design and that data were unavailable for 2009, the year prior to our analysis. However, future longitudinal data analyses arising from ACCEPt will investigate the impact of chlamydia test results on future GP testing performance.

We found that during 2010, chlamydia testing rates were 3.7% among those aged 16 to 29 years, much lower than the 8.9% previously reported for Australian general practice [7]. This is likely due to the fact that we report here testing rates per GP rather than per clinic, as we are examining the associations between individual GP characteristics, knowledge and testing rates and patients may attend multiple different GPs at different times within each clinic. Patients who attended in 2010 would have only been eligible for one chlamydia test across the clinic, instead of a chlamydia test with each GP they consulted. When we examined chlamydia testing rates per clinic in our dataset, we found a chlamydia testing rate of about 10% per year, comparable to the earlier report [7].

In this study, only 21.2% of GPs identified the ideal follow-up interval following a positive chlamydia test as three months, with 56.3% reporting that they would re-test before 3 months. Similarly, Heal and colleagues surveyed a small group of GPs in northern Queensland and found that 54% of GPs re-tested at six weeks [27], although test of cure after treatment is not recommended over concerns about non-viable DNA being detected up to 4 weeks after treatment [28], and a test of re-infection at three months is instead advised. Inconsistent recommendations on follow-up testing after a positive result from the RACGP guidelines (3-12 months) [6] and the National Management Guidelines for STIs (three months) [29] may be a source of confusion. This highlights the need for consensus in guideline documents across Australia and national leadership from the Australasian Sexual Health Alliance [30] in promoting the new management guidelines released in August 2014 to all Australian GPs.

It was a concern that only about 40% of GPs correctly answered that one gram azithromycin is the recommended first line treatment for chlamydia infection during pregnancy, as untreated chlamydia can have consequences on pregnancy and neonates [31]. In the multivariable model that excluded GP characteristics, we found that GPs with correct knowledge about treatment during pregnancy had lower chlamydia testing rates. This is difficult to interpret and probably occurred as an artefact because there was some evidence that the male GPs in our sample did have better knowledge about correct treatment during pregnancy than females (43% versus 36%, p = 0.16), and male GPs also had lower testing rates on average compared with female GPs.

The higher chlamydia testing rates observed among metropolitan GPs requires further discussion and raises issues of equity. Firstly, people living in rural areas do have reduced access to primary care compared with those living in metropolitan areas. There are fewer GPs per capita in rural areas, patients have to wait longer for a consultation [32] and there are fewer females GPs available [33]. This contributes to reduced possibilities for chlamydia testing among those living in rural areas which may lead to higher chlamydia prevalence in rural areas as recently confirmed in a survey of men and women aged 16-29 years attending general practice that found chlamydia prevalence was slightly higher in rural areas compared to metropolitan areas (4.8% versus 3.1%, p = 0.08) [34]. Secondly, there are issues for young people seeking sexual health care treatment in rural areas with young people reporting fear, embarrassment or shame where `everyone knows each other’ [35]. However, we recently found in a survey of 3724 men and women aged 16 to 29 years living in rural Australia, 70% will agree to have a chlamydia test when offered by their GP [34]. This suggests that young people may not actively seek a test, but will accept one if asked by their GP.

## Conclusion

We have linked GP chlamydia testing rates with GP’s knowledge of chlamydia and found that testing rates are higher among female GPs, those working in metropolitan areas, those with an interest in sexual health and among those with greater knowledge of the RACGP chlamydia testing guidelines while lower testing rates were associated with older GPs. These findings highlight the need for improved quality and effectiveness of sexual health education for GPs, particularly male GPs and given the disparities in health care access between metropolitan and rural areas, this is urgently needed if we are to address potential equity issues around sexual health service access.

## Authors’ contributions

AY conducted the data analysis and drafted the manuscript. MTS helped to draft the manuscript. SS carried out the data extraction. ML directed the statistical analysis. AW participated in data collection. KS designed the questionnaires. RG, CKF, BD, JK, JG, MP and RC had input on the study design and provided feedback on the analysis and the manuscript. JH conceived of the study, directed the analysis and helped to draft the manuscript. All authors read and approved the final manuscript.

## Authors’ original submitted files for images

Below are the links to the authors’ original submitted files for images. Authors’ original file for figure 1

## Abbreviations

GP: General practitioner
PID: Pelvic inflammatory disease
RACGP: Royal Australian College of General Practitioners
