# Supplementary material for: Improving chlamydia knowledge should lead to increased chlamydia testing among Australian general practitioners: a cross-sectional study of chlamydia testing uptake in general practice
Source: BMC Infect Dis. 2014 Nov 7;14:584. doi: 10.1186/s12879-014-0584-2 (PMC4228271; doi:10.1186/s12879-014-0584-2)
Supplement: Supplementary file 1 — Authors’ original file for figure 1 [file 12879_2014_584_MOESM1_ESM.doc]

Table 1: Characteristics of participating GPs

| Characteristic |  | Overall  N (%) | Male GP N (%) | Female GP N (%) | p-value |
| --- | --- | --- | --- | --- | --- |
| Gender of GP |  |  | 258 (66.0) | 133 (34.0) | <0.01 |
| Location of GP clinic | Rural | 346 (88.5) | 234 (90.7) | 112 (84.2) | 0.06 |
|  | Metropolitan | 45 (11.5) | 24 (9.3) | 21 (15.8) |  |
| GP Age Group (years) | <30 | 13 (3.3) | 6 (2.3) | 7 (5.3) | <0.01 |
|  | 30-44 | 124 (31.7) | 70 (27.1) | 54 (40.6) |  |
|  | 45-59 | 207 (52.9) | 143 (55.4) | 64 (48.1) |  |
|  | >60 | 47 (12.0) | 39 (15.1) | 8 (6.0) |  |
| Years working in general practice | <5 | 74 (19.2) | 41 (16.1) | 33 (25.0) | <0.01 |
|  | 5-10 | 40 (10.4) | 18 (7.1) | 22 (16.7) |  |
|  | 10-20 | 89 (23.1) | 57 (22.4) | 32 (24.2) |  |
|  | 20-30 | 117 (30.3) | 83 (32.7) | 34 (25.8) |  |
|  | 30+ | 66 (17.1) | 55 (21.7) | 11 (8.3) |  |
| Country of medical training | Australia | 247 (63.3) | 161 (62.7) | 86 (64.7) | 0.70 |
|  | Overseas | 143 (36.7) | 96 (37.4) | 47 (35.3) |  |
| Number of clinic sessions per week | <5 | 35 (9.2) | 11 (4.4) | 24 (18.5) | <0.01 |
|  | 5-9 | 267 (70.3) | 176 (70.4) | 91 (70.0) |  |
|  | 10+ | 78 (20.5) | 63 (25.2) | 15 (11.5) |  |
| Postgraduate qualifications^1^ | No | 117 (29.9) | 75 (29.1) | 42 (31.6) | 0.61 |
|  | Yes | 274 (70.1) | 183 (70.9) | 91 (68.4) |  |
| Interest in sexual health | No | 284 (73.8) | 207 (80.9) | 77 (59.7) | <0.01 |
|  | Yes | 101 (26.2) | 49 (19.1) | 52 (40.3) |  |

1. Qualifications include Diploma of Obstetrics & Gynaecology, Diploma of Venereology/Sexual Health, Certificate of the Family Planning Association, Fellow of the Royal Australian and New Zealand College of Obstetricians and Gynaecologists, Fellow of the Royal Australian College of General Practitioners, Fellow of the Australian College of Rural and Remote Medicine

Table 2: Knowledge about chlamydia diagnosis and management by GP gender

| Variable |  | Overall  N (%) | Male GP N (%) | Female GP N (%) | p-value |
| --- | --- | --- | --- | --- | --- |
| Female age groups at highest risk of infection^1^ | Incorrect | 24 (6.3) | 16 (6.4) | 8 (6.1) | 0.91 |
|  | Correct | 244 (93.8) | 236 (93.7) | 124 (93.9) |  |
| Male age groups at highest risk of infection^2^ | Incorrect | 39 (10.4) | 24 (9.8) | 15 (11.5) | 0.63 |
|  | Correct | 336 (89.6) | 220 (90.2) | 116 (88.6) |  |
| Chlamydia is usually asymptomatic in Women | Disagree | 32 (8.3) | 20 (7.9) | 12 (9.0) | 0.70 |
|  | Agree | 355 (91.7) | 234 (92.1) | 121 (91.0) |  |
| Chlamydia is usually asymptomatic in Men | Disagree | 100 (26.0) | 67 (26.6) | 33 (24.8) | 0.71 |
|  | Agree | 285 (74.0) | 185 (73.4) | 100 (75.2) |  |
| Knowledge of population groups to be targeted for screening^3^ *(see below for results for each scenario)* | 0-2 | 24 (6.2) | 19 (7.5) | 5 (3.8) | 0.09 |
|  | 3-5 | 264 (68.6) | 177 (70.2) | 87 (65.4) |  |
|  | 6+ | 97 (25.2) | 56 (22.2) | 41 (30.8) |  |
| Treatment in men and non-pregnant women^4^ | Incorrect | 70 (17.9) | 49 (19.0) | 21 (15.8) | 0.43 |
|  | Correct | 321 (82.1) | 209 (81.0) | 112 (84.2) |  |
| Treatment in pregnant women^4^ | Incorrect | 231 (59.1) | 146 (56.6) | 85 (63.9) | 0.16 |
|  | Correct | 160 (40.9) | 112 (43.4) | 48 (36.1) |  |
| Retest 12 months after a negative test^5^ | Incorrect | 341 (87.2) | 233 (90.3) | 108 (81.2) | 0.01 |
|  | Correct | 50 (12.8) | 25 (9.7) | 25 (18.8) |  |
| Retest 3 months after a positive test^5^ | Incorrect | 308 (78.8) | 207 (80.2) | 101 (75.9) | 0.33 |
|  | Correct | 83 (21.2) | 51 (19.8) | 32 (24.1) |  |
| Knowledge of symptoms suggestive of PID^6^ | Incorrect | 109 (27.9) | 83 (32.2) | 26 (19.6) | 0.01 |
|  | Correct | 282 (72.1) | 175 (67.8) | 107 (80.5) |  |
| Knowledge of PID tests that should be done^7^ | Incorrect | 205 (52.4) | 148 (57.4) | 57 (42.9) | 0.01 |
|  | Correct | 186 (47.6) | 110 (42.6) | 76 (57.1) |  |
| Testing Scenarios |  |  |  |  |  |
| Case 1: 23 year old female, pap smear | Not offer test | 180 (46.3) | 136 (53.1) | 44 (33.1) | <0.01 |
|  | Offer test | 209 (53.7) | 120 (46.9) | 89 (66.9) |  |
| Case 2: 18 year old female, abdominal pain | Not offer test | 19 (4.9) | 16 (6.2) | 3 (2.3) | 0.08 |
|  | Offer test | 371 (95.1) | 241 (93.8) | 130 (97.7) |  |
| Case 3: 26 year old male, truck license medical | Not offer test | 315 (80.8) | 217 (84.4) | 98 (73.7) | 0.01 |
|  | Offer test | 75 (19.2) | 40 (15.6) | 35 (26.3) |  |
| Case 4: 24 year old female, 16/40 pregnant | Not offer test | 228 (58.8) | 151 (59.2) | 77 (57.9) | 0.80 |
|  | Offer test | 160 (41.2) | 104 (40.8) | 56 (42.1) |  |
|  | | | | | |
| Case 5: 22 year old male, Aboriginal, sore throat | Not offer test | 271 (69.5) | 178 (69.3) | 93 (69.9) | 0.89 |
|  | Offer test | 119 (30.5) | 79 (30.7) | 40 (30.1) |  |
| Case 6: 33 year old female, pill script | Not offer test | 330 (84.8) | 225 (87.9) | 105 (79.0) | 0.02 |
|  | Offer test | 59 (15.2) | 31 (12.1) | 28 (21.1) |  |
| Case 7: 17 year old male, genital warts | Not offer test | 12 (3.1) | 11 (4.3) | 1 (0.8) | 0.06 |
|  | Offer test | 378 (96.9) | 246 (95.7) | 132 (99.3) |  |
| Case 8: 34 year old male, 2 partners in last 6 months, HIV test | Not offer test | 10 (2.6) | 6 (2.3) | 4 (3.0) | 0.70 |
|  | Offer test | 379 (97.4) | 250 (97.7) | 129 (97.0) |  |

1. Answers were classified as correct if they ticked at least one correct answer - 15-19 and 20-24 year olds; 2. Answers were classified as correct if they ticked at least one correct answer - 20-24 and 25-29 year olds; 3. Knowledge of population groups to be targeted for screening is one point for correctly offering a test under the 2010 RACGP guidelines – tests should be offered to cases 1, 2, 4, 5, 7 & 8 but not offered to cases 3 & 6; 4. Azithromycin is the correct treatment for men, women and pregnant women; 5. RACGP guidelines recommend re-testing every 12 months after a negative test and every 3 months after a positive test; 6. Correct signs and symptoms suggestive of PID are tenderness with motion of the cervix, adnexal tenderness, uterine tenderness, lower abdominal tenderness and inflamed cervix; 7. Correct diagnostic tests are pregnancy test, chlamydia and gonorrhoea test, abdominal palpation and bimanual examination

Table 3: GP characteristics and knowledge variables associated with chlamydia testing^1^

| GP Variable |  | Odds Ratio | 95%CI | p-value | Adjusted OR^2^ (including GP characteristics) | 95%CI | p-value | Adjusted OR^2^ (excluding GP characteristics) | 95%CI | p-value |
| --- | --- | --- | --- | --- | --- | --- | --- | --- | --- | --- |
| Gender of GP | Male  Female | 1.0 3.1 | 2.1, 4.7 | <0.01 | 1.0 2.5 | 1.9, 3.3 | <0.01 |  |  |  |
| Location | Rural  Metro | 1.0  2.3 | 1.4, 3.6 | <0.01 | 1.0  3.2 | 2.4, 4.3 | <0.01 |  |  |  |
| Age Group | <30  30-44  45-59  60+ | 1.0  0.8  0.6  0.4 | 0.5, 1.2  0.3, 0.9  0.1, 1.3 | 0.27  0.02  0.13 | 1.0  0.7  0.5  0.4 | 0.5, 1.1  0.3, 0.7  0.3, 0.7 | 0.13  <0.01  <0.01 |  |  |  |
| Years in general practice | <5  5-10  10-20  20-30  30+ | 1.0  1.0  0.9  0.8  0.4 | 0.6, 1.7  0.6, 1.5  0.4, 1.5  0.1, 1.3 | 0.91  0.81  0.48  0.11 |  |  |  |  |  |  |
| Country of training | Australian trained  Overseas trained | 1.0  0.8 | 0.5, 1.2 | 0.31 |  |  |  |  |  |  |
| Interest in sexual health | No  Yes | 1.0  1.7 | 1.0, 2.7 | 0.04 | 1.0 1.3 | 1.0, 1.7 | 0.03 |  |  |  |
| Postgraduate qualifications | No Yes | 1.0 1.2 | 0.7, 1.9 | 0.56 |  |  |  |  |  |  |
| Female age groups at highest risk of infection^3^ | Incorrect  Correct | 1.0  1.8 | 1.0, 3.1 | 0.05 | 1.0  1.6 | 0.7, 3.5 | 0.26 | 1.0  1.5 | 0.7, 3.5 | 0.31 |
| Male age groups at highest risk of infection^4^ | Incorrect  Correct | 1.0  1.6 | 0.9, 2.9 | 0.08 | 1.0  1.4 | 0.8, 2.7 | 0.23 | 1.0  1.6 | 0.9, 2.9 | 0.10 |
| Chlamydia is usually asymptomatic in Women | Disagree Agree | 1.0  1.4 | 0.9, 2.3 | 0.18 |  |  |  | 1.0  1.2 | 0.7, 2.1 | 0.44 |
| Chlamydia is usually asymptomatic in Men | Disagree Agree | 1.0 1.6 | 1.1, 2.2 | 0.01 | 1.0  1.4 | 1.0, 1.9 | 0.03 | 1.0  1.4 | 0.9, 1.9 | 0.10 |
| Knowledge of population groups to be targeted for screening^5^ | 0-2  3-5  6+ | 1.0  2.0  3.4 | 1.0, 4.0  1.7, 6.8 | 0.05  <0.01 | 1.0  1.7  2.0 | 0.8, 3.8  0.9, 4.4 | 0.16  0.08 | 1.0  2.0  2.9 | 1.0, 4.2  1.4, 6.2 | 0.07  <0.01 |
|  | | | | | | | | | | |
| Treatment in men and non-pregnant women^6^ | Incorrect Correct | 1.0  1.0 | 0.4, 2.3 | 0.97 |  |  |  | 1.0  1.0 | 0.5, 2.2 | 0.92 |
| Treatment in pregnant women^6^ | Incorrect Correct | 1.0  0.7 | 0.5, 1.0 | 0.11 |  |  |  | 1.0  0.6 | 0.4, 0.9 | 0.01 |
| Retest at 12 months after a negative test^7^ | Incorrect Correct | 1.0  1.8 | 0.9, 3.5 | 0.08 | 1.0  1.2 | 0.9, 1.6 | 0.32 | 1.0  1.5 | 0.9, 2.6 | 0.16 |
| Retest at 3 months after a positive test^7^ | Incorrect Correct | 1.0  1.0 | 0.7, 1.5 | 0.97 |  |  |  | 1.0  0.9 | 0.6, 1.4 | 0.73 |
| Knowledge of symptoms suggestive of PID^8^ | Incorrect Correct | 1.0  1.3 | 0.7, 2.2 | 0.38 |  |  |  | 1.0  1.3 | 0.8, 2.0 | 0.34 |
| Knowledge of PID tests that should be done^9^ | Incorrect Correct | 1.0  1.5 | 0.9, 2.3 | 0.09 | 1.0  0.9 | 0.7, 1.2 | 0.41 | 1.0  1.1 | 0.8, 1.7 | 0.51 |
| Gender of patient^2^ | Male  Female | 1.0  2.3 | 1.7, 3.1 | <0.01 | 1.0  1.9 | 1.5, 2.4 | <0.01 | 1.0  2.2 | 1.7, 2.9 | <0.01 |

1. Accounted for repeated measures from individuals GPs; 2. The multivariable models have adjusted for patient gender; 3. Answers were classified as correct if they ticked at least one correct answer - 15-19 and 20-24 year olds; 4. Answers were classified as correct if they ticked at least one correct answer - 20-24 and 25-29 year olds; 5. Knowledge of population groups to be targeted for screening is one point for correctly offering a test under the 2010 RACGP guidelines – tests should be offered to cases 1, 2, 4, 5, 7 & 8 but not offered to cases 3 & 6; 6. Azithromycin is the correct treatment for men, women and pregnant women; 7. RACGP guidelines recommend re-testing every 12 months after a negative test and every 3 months after a positive test; 8. Correct signs and symptoms suggestive of PID are tenderness with motion of the cervix, adnexal tenderness, uterine tenderness, lower abdominal tenderness and inflamed cervix; 9. Correct diagnostic tests are pregnancy test, chlamydia and gonorrhoea test, abdominal palpation and bimanual examination.
